# Impact of Moderate Individually Tailored Physical Activity in Multiple Sclerosis Patients with Fatigue on Functional, Cognitive, Emotional State, and Postural Stability

**DOI:** 10.3390/brainsci11091214

**Published:** 2021-09-15

**Authors:** Justyna Redlicka, Ewa Zielińska-Nowak, Anna Lipert, Elżbieta Miller

**Affiliations:** 1 Department of Neurological Rehabilitation, Medical University of Lodz, Milionowa 14, 93-113 Lodz, Poland; justyna.redlicka@umed.lodz.pl (J.R.); ewa.zielinska@umed.lodz.pl (E.Z.-N.); 2 Department of Sports Medicine, Medical University of Lodz, Pomorska 251, 92-213 Lodz, Poland; anna.lipert@umed.lodz.pl

**Keywords:** multiple sclerosis, fatigue, moderate physical activity

## Abstract

Multiple sclerosis (MS) is a chronic disease, with fatigue syndrome as one of the main symptoms. The aim of this study was to demonstrate that moderate physical activity (MPA) may have a beneficial effect on postural stability, balance, and clinical parameters. The research group consisted of 137 randomized patients hospitalized at the Department of Neurological Rehabilitation, Medical University of Lodz. Finally, 76 patients were qualified who were divided into two groups—high fatigue (HF) and low fatigue (LF). Participants were assessed twice: before and after a 4-week MPA program using: the Expanded Disability Status Scale (EDSS), the Fatigue Severity Scale (FSS), the Mini-Mental State Examination (MMSE), the Montreal Cognitive Assessment (MoCA), the Beck Depression Inventory (BDI), and the Geriatric Depression Scale (GDS), and stabilometric platform tests were performed. Results obtained after the 4-week MPA program showed a positive effect of the MPA with differences between LF and HF groups. The MPA was more effective in MS patients with LF in cognitive functions, functional status, and postural stability but among HF patients in an emotional state, especially in MS patients below 65 years, although in total, both groups benefited from the MPA.

## 1. Introduction

Multiple sclerosis (MS) is a chronic heterogeneous disease with an unpredictable clinical course. Patients with MS experience a wide range of symptoms, such as paralysis, ataxia, spasticity, incontinence, and fatigue syndrome. Fatigue is reported in 70–80% of patients and is considered one of the most prevalent and disabling symptoms in all stages of the illness [1]. This common kind of lassitude is unique to people with MS. It is also called MS-related fatigue and is not associated with the level of disability or depression [2]. Specific features of MS-related fatigue are heat and humidity sensitivity, which highly interfere with daily functioning [3].

Chronic symptoms in CNS diseases, including fatigue, are a great challenge in everyday clinical practice. Currently, standard MS neurological drug disease-modifying therapies are concentrated on an early stage and focused on lowering the frequency of clinical relapses and decreasing the number of new lesions in magnetic resonance brain screenings [4]. Pharmacological treatment of fatigue is rather low-effective. The reasons are very complex. Firstly, the pathogenesis of fatigue is not entirely understood; secondly, the biochemical delivery to the brain is obstructed due to the blood–brain barrier [5] and is the most important. The process of diagnosis is based on self-report fatigue questionnaires, so it can be an under-recognized symptom [6]. There are several pharmacological compounds such as amantadine, modafinil, and 4-aminopyridine used as a part of complex therapy often used in combination with antidepressants [7] or other additional therapies such as cryostimulation, behavioral therapy, or aerobic training [8].

For many years, physicians have recommended avoiding physical activity due to the heat sensitivity among MS patients. Nowadays, accumulating data from clinical studies present the benefits of regular exercise, which can limit deconditioning and improve patients’ functional and mental status [9]. A recently published review of 26 randomized controlled trials (RCTs) on an exercise program in MS patients presented little decreased risk of relapse in active MS patients versus nonactive MS (6.3% versus 4.6%) [10]. Currently, the National Multiple Sclerosis Society recommends physical activity for people with MS (150 min per week or even more), preferably aerobic training and breathing exercises [11].

However, there is still a lack of clinical studies searching for a correlation between MS-related fatigue, moderate physical activity, and its potential clinical benefits [12].

Furthermore, little is known about the severity of fatigue syndrome and the differences between LF and HF levels among MS patients and their potential impact on functional, cognitive, and emotional status, as well as postural stability.

Therefore, this study compares MS patients with LF and HF and searches for the potential benefits of the moderate physical activity (MPA) program in these two study groups to specify recommendations for patients with MS-related fatigue.

From a clinical point of view, it is important to understand whether MPA has any specific correlation with a reduction in fatigue and to specify which group of patients (LF or HF) should be particularly targeted with this kind of training. Moreover, we hypothesized the potential benefits of MPA on postural stability and balance, as well as clinical parameters, including depression, cognition, and functional status.

## 2. Materials and Methods

### 2.1. Participants

**Figure 1:**
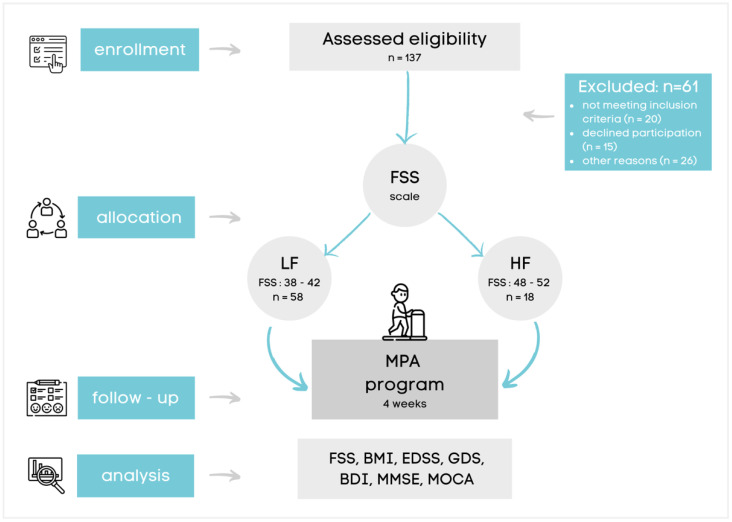
Process of the study.

This intervention was conducted according to TIDier guidelines. The research sample consisted of 137 randomized MS patients (ICD G35.0) hospitalized at the Neurological Rehabilitation Department of the Medical University of Lodz, Poland. We excluded 61 MS patients (20 not meeting inclusion criteria, 15 declined participation, and 26 showed other reasons). Finally, 76 MS patients were enrolled from a total of 137. Inclusion criteria for this study were: diagnosis of MS according to the McDonald criteria, age 18 years or older, EDSS score below 6, MMSE > 15 and MoCA > 10, diagnosis of fatigue syndrome based on FSS score over 38, lack of relapses during the previous 3 months, and not receiving an MPA training program in the last 6 months (Figure 1).

Moreover, our patients did not use any drugs recommended for the treatment of fatigue (such as amantadine, 4-aminopyridine, or modafinil) to avoid possible interferences with the antifatigue effect of MPA training. All eligible subjects received verbal and written information and provided written consent to participate in the study. The protocol and procedures were followed according to the Helsinki Declaration and were approved by the Ethics Committee of the Medical University of Lodz, Poland, RNN/168/21/KE. This study was conducted by the experienced rehabilitation team of our Neurological Rehabilitation Department (medical doctor—enrollment, supervisor; physiotherapist—MPA intervention, stabilometric assessment, estimation of EDSS scale; psychologist—estimation in psychological scales). The data were collected face to face. Both groups were examined at two stages: before and after the 4-week MPA program (Figure 1).

### 2.2. Clinical Scales

Background demographics included age, gender, MS diagnosis ICD (G 35.0), duration of illness (years since diagnosis), body mass index (BMI), and administration of antidepressants or muscle relaxants (Table 1).

Data for fatigue were collected using the Fatigue Severity Scale (FSS). This is a widely validated tool consisting of nine fatigue-related statements rated on a seven-point scale (disagree to agree). After performing the distribution of the results measured by FSS and calculating the *ceiling and floor effect*, we observed a bimodal distribution, which means that among the patients, we observed the two most common mean values, first in the range between 38 and 42 and then between 48 and 52 points. We used the value of 38 as a cut-off value, as suggested by Flachenecker et al. [13], to include only fatigued patients. Then, MS patients were divided into two subgroups depending on their level of fatigue. The first group, LF, presented an FSS score between 38 and 42, and the second group, HF, presented an FSS score between 48 and 52. There were 40 females and 36 males in both groups who completed the entire study: 58 participants with LF and 18 with HF. Both groups are described in Table 1. Functional state was assessed using the EDSS [14]. The EDSS is the most commonly used scale for the assessment of impairment and disability. Cognitive functioning and depression were assessed in two different clinical scales depending on patients’ age. Therefore, cognition was assessed with the Mini-Mental State Examination (MMSE) in MS patients over 65 years old and with the Montreal Cognitive Assessment (MoCA) below the age of 65. Both scales are the most widely used screening tests in people with CNS diseases, including MS, and provide a brief and objective measure of cognitive functioning. MMSE (0–30) score ≥27 indicates normal cognitive functioning, 18–26 mild cognitive impairment, and 0–17 severe cognitive impairment [15,16]. MoCA (0–30) score ≥26 indicates no cognitive impairment [17].

In terms of depression, the Geriatric Depression Scale (GDS) assessed patients over 65 years old and the Beck Depression Inventory (BDI) below the age of 65. Both of these scales are valid self-report screening tools for measuring depression in the elderly, including MS patients [18]. GDS consists of 30 yes/no questions scored 0 or 1 points. A cut-off of ≥21 indicates severe depression, 11–20 mild depression, and ≥10 no depression [19].

The Beck Depression Inventory (BDI) is a 21-question questionnaire with a cut-off score of ≥11 meaning no depression, 12–26 indicating mild depression, 27–49 moderate–severe depression, and 50–63 severe depression [18].

### 2.3. Stabilometric Assessment

Postural stability and body balance assessment were carried out by stabilometric estimation with the CQ-Stab 2-platform posturograph (CQ Electronic System). Before examination, the platform plates were aligned (two 30 s tests). Next, the postural stability assessment was perfumed in a standing position with eyes wide open (EO) and closed eyes (EC). The width and angle between legs were natural and not forced. A fixation point was located at a distance of 1 m opposite the participant. The examined person stood on a platform with strain gauges placed in the corners recording the central pressure of the feet on the ground (reflecting the projection of the center of gravity onto the base plane), as well as its displacements in the sagittal *x*-axis, i.e., left–right and frontal Y, i.e., front–back. The projection of the center of pressure of the feet on the ground was therefore recorded as a point and as a dynamic parameter changing the position in a unit of time. The statokinesiometric tests were performed with eyes open and then with eyes closed. The results of the study are presented in the form of graphs called a statokinesiogram and stabilogram. The statokinesiogram shows the movement of the pressure center in a coordinate system where the *x*-axis corresponds to the snares in the right–left direction and the *y*-axis to the snares in the anterior–posterior direction. The stabilogram shows the location of the pressure center as a function of time, with movement in the right–left direction and in the anterior–posterior directions separately.

Subjects were told to stay as still as possible. The operator stood behind the subject to prevent falls. Accuracy and reliability of the data have been provided elsewhere. Clinical and instrumental assessment at baseline was carried out in one session. After 4 weeks of the MPA rehabilitation program, the estimations were repeated, and subjects were asked to wear the same shoes as worn during the baseline assessment.

### 2.4. Moderate Physical Activity (MPA)

**Figure 2:**
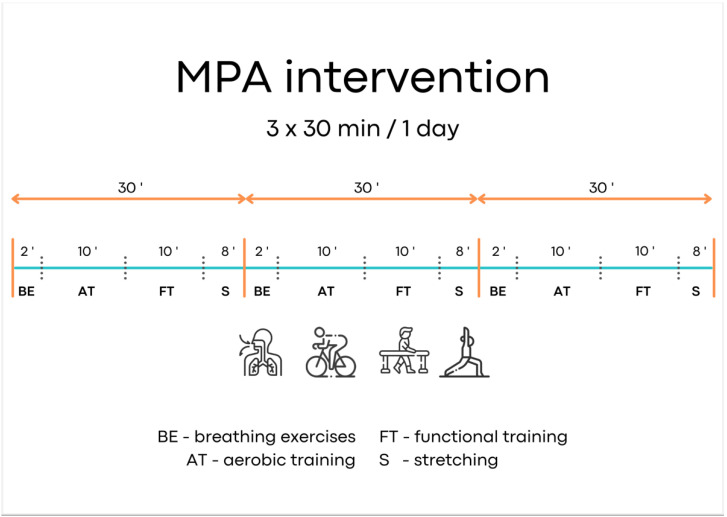
MPA intervention per day.

After the baseline measurements, both groups started the supervised MPA program, with individual training tailored to the needs and possibilities of the patient. MS patients participated in 6 sessions per week for 4 weeks. Training sessions were interspersed by one day of rest (Sunday) to maintain proper recovery. Each session started with 2 min of breathing exercises. Next, the first part of MPA (10 min) consisted of aerobic training on a cycloergometer, and the second part (10 min) comprised functional training, which was individually tailored and adjusted to patients’ needs (walking; balance workout; muscle strength; climbing the stairs, etc.). After the MPA, 8 min stretching exercises and relaxation therapy were performed (Figure 2).

Intensity of MPA was personalized, and patients’ heart rate was monitored to reach a specific range (50–70%) of maximal heart rate in relation to individual capabilities. The second part of MPA was individually tailored and adjusted to patients’ needs. The MPA per day lasted 90 min (3 × 30 min) and was performed at a medium workload corresponding to 12–14 ratings of perceived exertion on a 20-point Borg scale (RPE). The Borg Rating of Perceiver Exertion Scale evaluates perceived exertion and is a good tool to assess the intensity of training. The scale range is from 6 (no exertion) to 20 (maximal physical effort) [20]. A regular increase in training load (from 12 to 14 RPE) over the 4 weeks of MPA training was performed. At the end of the MPA, stretching of the extremities and RPE level were monitored. There were no modifications during the course of the study.

### 2.5. Statistical Analysis

Statistical analyses were performed using Statistical version 13.1 software (StatSoft, Tulsa, OK, USA). The data were not normally distributed, so nonparametric tests were used. Statistical analysis was performed using the Welch test for dependent variables and the Mann–Whitney U-test *t* for independent variables. The effect size measure of differences between the results from the beginning and after MPA rehabilitation inside the groups or the results between the LF and HF groups was verified by Cohen’s *d* test, defined as the difference between two means divided by a standard deviation for the data. Cohen’s test classified effect sizes as small (*d* = 0.2), medium (*d* = 0.5), and large (*d* ≥ 0.8). Spearman correlation was used to assess the relationship between: (1) the changes in the results of BDI, MOCCA, and the stabilometric platform for patients below 65 years; (2) the changes in the results of GDS, MMSE, and the stabilometric platform for patients over 65 years. Significant differences were accepted for all analyses at the level of *p* < 0.05.

## 3. Results

### 3.1. MPA and Emotional Status

Generally, the results obtained after MPA showed a positive change in the emotional status of the MS patients. The most positive effect was observed in HF patients (BDI below 65 years) (0.001; medium effect size) in comparison with BDI in the LF group (0.05; small effect size). The estimation in GDS in the group of MS patients over 65 years showed a medium effect size in HF and a small effect size in LF without statistically significant changes in both groups (Table 2).

### 3.2. MPA and Cognitive Function

Only in the LF group were statistically significant changes in cognitive functioning after MPA observed. In the group of patients with age below 65 years, estimation in MoCA showed statistically significant changes (0.001) with a medium effect size. Estimation of MS patients over the age of 65 assessed in MMSE also showed statistically significant changes (0.001) with a small effect size. In the group of HF MS, patients there was a small effect size without statistical significance (Table 2).

### 3.3. MPA and Functional State

In the whole group of MS-fatigued patients, there were improvements in functional estimations in EDSS after MPA (0.001; small effect size). However, the results of LF patients were statistically more significant (0.001; small effect size) vs. the HF group (0.05; small effect size). There were no statistically significant differences in functional status between patients with LF and HF regardless of the age of the patients (Table 2).

### 3.4. MPA and Stabilometric Evaluation

The results obtained using the stabilometric platform improved after MPA (Table 3).

Statistically significant differences in the results were observed in the mean deflection of the center of pressure of the feet from Point 0 in the direction of the *y*-axis in millimeters with eyes open (MAAP-EO) (LF-0.0001; medium effect size vs. 0.102; medium effect size), the mean deflection of the center of pressure of the feet from Point 0 in the direction of the *x*-axis in millimeters with eyes opened (MAML-EO) (LF-0.008 vs. HF 0.024; medium effect size), the mean deflection of the center of pressure of the feet from Point 0 in the direction of the *y*-axis in millimeters with eyes closed (MAAP-EC) (LF-0.0001; medium effect size vs. HF 0.009; small effect size), and the mean deflection of the center of pressure of the feet from Point 0 in the direction of the *x*-axis in millimeters with eyes closed (MAML-EC) in patients with LF (0.0002; small effect size). In patients with HF, statistically significant differences in the results were observed in MAML-EO and MAAP-EC (Table 3).

### 3.5. Spearman Correlation

There were no statistically significant differences in functional status between patients with LF and HF regardless of the age of patients. Moreover, there was no statistically significant correlation between EDSS scale and high levels of fatigue compared with people with low levels of fatigue. The Spearman test showed the relationship between the results of the stabilometric platform and emotional status, as well as cognitive function. Among participants below 65 years with LF, statistically significant data were observed in correlation with BDI and MAML-EO (*p* = 0.288), and with MAML-EC (*p* = 0.301), similar associations were noticed, but the results of the correlations were not statistically significant in HF patients (Table 4).

Among participants over the age of 65 with HF, some correlations were noticed between the results obtained in GDS and MMSE tests and the results of stabilometric platform; however, the existing associations were not statistically significant (Table 5).

## 4. Discussion

This study was developed to analyze whether moderate individually tailored physical activity has any impact on the postural stability, functional status, cognitive impairment, and emotional status of MS patients with fatigue syndrome. Moreover, we divided our participants into LF and HF groups using the FSS scale. In our previous research investigating cryostimulation as an additional therapy for fatigue in MS patients, we observed differences in the effectiveness of treatment between these two groups with better clinical effect in feeling of fatigue and functional status in HF patients [8]. Generally, fatigue is a very common problem in MS and can occur at all stages of the disease. There are two kinds of MS-related fatigue: primary fatigue, associated with disease process (correlated with demyelination and axonal loss in the CNS); and secondary fatigue, associated with MS complications, such as sleep disorders, reduced activity, and depression [21]. Recently, nonpharmacological strategies have been introduced for the treatment of patients with MS-related fatigue, including physical exercise (aerobic work), cryostimulation, energy conservation strategies, and cognitive behavioral therapy [22]. Accumulating data suggest that regular physical activity is strongly recommended in MS patients with fatigue [23,24]. Lack of physical activity in MS patients can accelerate potential complications, such as deconditioning, osteoporosis, obesity, or cardiological problems; however, regular training might be an important factor of neuroprotection [25].

Our study shows that the 4-week MPA program has a positive impact on balance, postural stability, functional status, cognition, and emotional status. This might be the result of the significant and large fatigue decrease observed in both groups. A significant positive change in the level of depression after MPA estimated in BDI was observed in the group of 53 MS patients below the age of 65 and was much more significant in patients with high rather than low fatigue. These findings are very important, because depression is one of the major problems in MS, which may affect up to 50% of patients [26], significantly impacting other symptoms such as fatigue and pain and negatively affecting cognition and quality of life.

A more recent review of 13 RCTs on the effect of exercise on depressive symptoms in MS patients reported an effect size of 0.36 [27]. Moreover, depression might be a side effect of standard neurological treatment with steroids or interferon. Accumulated data present the positive impact of physical activity intervention on fatigue and depression in MS [28,29]. Cognitive decline reduces the everyday activity of MS patients and can have a great impact on quality of life [30].

Our research presents a significant positive effect of a 4-week MPA program on cognitive functioning in a group of 58 LF MS patients, which suggests that MPA should be recommended as early as possible, even for MS patients with a lower level of fatigue. However, our inclusion criteria were MMSE >15 and MoCA >10; therefore, the reason for this improvement might partially be a rather low baseline score.

The present study also demonstrated that the level of disability (EDSS) decreased in MS patients with low fatigue after MPA; however, the results of MS patients below 65 years were more significant.

There were no statistically significant differences in functional status between patients with LF and HF regardless of the age of patients. Moreover, there was no statistically significant correlation between EDSS scale and HF and LF levels. These results indicate that the neurologic status of individuals with MS may not adequately predict the severity of fatigue experienced by them. These findings are consistent with previous work by Bakshi et al., who found no significant relationship between fatigue (measured by the Fatigue Severity Scale) and EDSS score prospectively in a large sample of MS patients [31]. Krupp et al. also demonstrated no significant associations between fatigue severity (measured by the visual analog scale) and EDSS score in people with MS [32]. On the other hand, Rasova et al. observed a better reaction to aerobic training among patients with an EDSS score ≤3 with significant improvements not only in fatigue and depression but also in muscle performance, pulmonary ventilation, and perception of effort; however, among patients with an EDSS score between 3 and 6.5, there has also been a tendency to improve in these parameters [33]. There are many studies that indicate a positive impact of exercise training on fatigue in MS patients, but most of them focus on patients with mild or moderate level of disability according to EDSS score [34,35]. However, there are also some promising data among patients with severe disabilities [36].

In a study by Garg et al., patients with higher levels of fatigue (MFIS > 10) were also associated with greater functional mobility impairment, increased depression, and decreased quality of life in comparison to the group with lower fatigue levels (MFIS ≤ 10). Only neurological deficit (measured by EDSS) was similar in both groups [37].

Another very important problem in MS patients’ everyday life is gait disturbance. Over 80% of MS patients pointed them out as their main complaint [38]. Our study presents statistically significant differences in MAAP-EO, MAML-EO, MAAP-EC, and MAML-EC in patients with LF. In patients with HF, statistically significant differences in the results were observed in MAML-EO and MAAP-EC. This suggests that fatigue has an impact on balance and postural stability. This is consistent with a previous study by Hebert et al., in which symptomatic fatigue was found to be related to balance, and was a predictor of balance as a function of the central sensory cortex among MS patients [39]. On the other hand, a vestibular rehabilitation program consisting of balance exercises and eye movement exercises significantly improved not only balance but also fatigue, which may indicate that balance disturbances may also negatively influence perception of fatigue [40,41].

Altogether, MPA has a positive effect on MS patients with fatigue, with significant differences between LF and HF groups. MPA training was more effective in the HF group in emotional status and in the LF group in cognitive functions in MS patients with age below 65. Balance and postural stability were improved in four variables in the LF group and in two variables in the HF group. Symptomatic fatigue is related to balance and is a possible predictor of balance dysfunction in people with MS. Fatigue and balance are associated with cerebellar and brainstem involvement [39].

This study provides early evidence supporting the theory that for MS patients with fatigue who struggle to maintain the ability to cope with the MPA of everyday tasks, training is a good solution, especially for MS patients below 65 years.

## 5. Limitations and Strengths of the Study

There are some limitations that should be included for data interpretation. The main limitation was the small number of MS patients, as well as an unbalanced number of participants in groups, who were enrolled in the study; however, it was still relatively large in comparison to previous studies analyzing a similar area of interest. Moreover, another limitation was that this study included male and female participants, as well as participants with different strength levels, which may have impacted the results [42].

Additionally, this study included people with an EDSS below 6; therefore, the results are characteristic to a defined level of disability, and the recommendations are targeted at patients with small and medium levels of disability.

## 6. Conclusions

This study compares MS patients with low and high levels of fatigue and searches for the potential benefits of the moderate physical activity program in these two study groups to specify recommendations for patients with MS-related fatigue. The main strength of this study is comparing MS patients between two groups, depending on the level of fatigue, as well as potential practical clinical aspects. Fatigue negatively influences other MS symptoms such as balance disturbances, reduced postural stability, cognitive functioning, depression, and general quality of life. Proper management of fatigue, adjusted to its level, may be beneficial in these aspects; therefore, it is important to include fatigue assessment in MS patients’ evaluation. There is a constant need to emphasize the importance of nonpharmacological treatment of fatigue among MS patients.

## Figures and Tables

**Table 1:** Baseline characteristics of fatigued multiple sclerosis patients with high fatigue (HF) and low fatigue (LF).

	HF (*n* = 18)	LF (*n* = 58)	Total (*n* = 76)
Gender (Male/Female)	9/9	27/31	36/40
Age (Years)	63.50 (53.83–65.17)	57.00 (50.73–57.89)	60.00 (52.51–58.57)
Body Height (cm)	168.50 (163.45–175.11)	170.00 (167.82–172.22)	169.50 (167.74–171.94)
Body Mass (kg)	79.90 (70.39–87.64)	75.15 (72.29–79.39)	76.00 (73.29–79.89)
BMI (kg/m^2^)	25.50 (22.17–29.26)	26.00 (24.99–26.96)	26.00 (24.84–26.99)
MS Duration (years)	10.72 (7.8–9.62)	11.2 (6.43–8.69)	10.96 (7.11–9.15)
MS Type (RR/SP)	19/26	21/10	40/36
Antidepressants (no. of patients)	7	5	12
Muscle relaxants (no. of patients)	36	9	45

HF: high fatigue; LF: low fatigue; BMI: body mass index; MS: multiple sclerosis; RR: relapsing remitting; SP: secondary progressive.

**Table 2:** Characteristics of the influence of MPA on emotional status (BDI and GDS), cognitive functions (MMSE and MOCCA), and functional state (EDSS) before and after MPA.

	All			LF			HF		
	Before	After	Changes	Before	After	Changes	Before	After	Changes
EDSS (↑ 65 years)	6.38 ± 0.71	6.07 ± 0.55 ***^M^	0.28 ± 0.30 (0.15–0.42)	6.35 ± 0.69	6.04 ± 0.52 **^M^	0.31 ± 0.25 (0.15–0.46)	6.37 ± 0.79	6.12 ± 5.59	0.25 ± 0.38 (−0.06–0.56)
EDSS (↓ 65 years)	5.60 ± 1.38	5.34 ± 1.36 ***^S^	0.25 ± 0.33 (0.16–0.34)	5.70 ± 1.37	5.42 ± 1.35 ***^S^	0.28 ± 0.33 (0.18–0.38)	5.15 ± 1.41	5.00 ± 1.39	0.15 ± 0.34 (−0.09–0.39)
GDS (↑ 65 years)	4.19 ± 7.20	3.62 ± 5.83 ^S^	0.57 ± 4.06 (0.97–6.27)	6.15 ± 8.57	5.69 ± 6.63 ^S^	0.46 ± 5.11 (−2.62–3.55)	1.00 ± 1.85	0.25 ± 0.71 ^M^	0.75 ± 1.48 (−0.49–1.99) ^S^
BDI (↓ 65 years) *n* = 53	8.60 ± 8.08	6.34 ± 6.87 ***^M^	2.25 ± 4.45 (1.05–3.46)	8.47 ± 8.36	6.62 ± 7.16 *^S^	1.84 ± 4.70 (0.43–3.26)	9.20 ± 7.07	5.10 ± 5.51 ***^M^	4.10 ± 2.47 (2.33–5.87) ^M^
MMSE (↑ 65 years)	21.20 ± 5.87	23.20 ± 5.96 *^S^	−0.95 ± 2.22 (−1.96–0.06)	21.85 ± 7.30	25.40 ± 2.60 ***^S^	−1.00 ± 2.58 (−2.56–0.56)	19.62 ± 8.13	20.15 ± 2.22 ^S^	2.39 ± 0.74 (1.77–3.01)
MOCA (↓ 65 years) *n* = 53	20.36 ± 8.07	23.13 ± 8.85 ***^M^	−2.76 ± 2.62 (−3.47–−2.05)	20.62 ± 8.08	23.62 ± 8.83 ***^M^	−3.00 ± 2.26 (−3.68–−2.32)	19.20 ± 8.36	20.90 ± 9.06 ^S^	−1.70 ± 3.86 (−4.46–1.06) ^M^

EDSS: Expanded Disability Status Scale; GDS: Geriatric Depression Scale; BDI: Beck Depression Inventory; MMSE: Mini-Mental State Examination; MOCA: Montreal Assessment Cognitive. Significant differences between the results obtained “before” and “after” MPA rehabilitation: * *p* < 0.05; ** *p* < 0.01; *** *p* < 0.001. Significant differences between changes were observed in the LF and HF groups when *p* < 0.05. The letters L, M, and S indicate large, moderate, and small effect sizes, respectively. ↑: the group of MS patients over 65 years. ↓: the group of patients with age below 65 years.

**Table 3:** The influence of MPA on MS patients with LF and HF using a stabilometric platform.

	LOW FATIGUE Group (LF)			HIGH FATIGUE Group (HF)		
	Before MPA	After MPA	*p*	Before MPA	After MPA	*p*
MAAP-EO	4.25 (4.08–5.74)	3.05 (2.94–4.33)	0.0001 ^M^	5.35 (3.81–9.11)	3.30 (3.15–6.42)	0.102 ^M^
MAML-EO	2.30 (2.90–5.41)	2.10 (2.48–4.90)	0.008	3.20 (2.47–6.98)	2.35 (1.84–4.52)	0.024 ^M^
MDDB-EO left leg	49.00 (45.84–50.88)	48.50 (47.67–51.63)	0.401	47.50 (44.07–52.26)	47.50 (44.64–51.80)	0.887
MDDB-EO right leg	51.00 (49.12–54.16)	51.50 (48.35–52.31)	0.399	52.50 (47.73–55.93)	52.50 (48.20–55.36)	0.887
MAAP-EC	3.90 (3.96–5.76)	2.40 (2.67–4.28)	0.0001 ^M^	4.00 (3.03–10.10)	2.90 (2.13–7.66)	0.009 ^S^
MAML-EC	1.90 (2.25–4.14)	1.50 (1.46–4.61)	0.0002 ^S^	2.40 (1.04–8.56)	1.50 (0.66–7.27)	0.108 ^S^
MDDB-EC left leg	49.00 (46.25–51.65)	49.00 (47.58–51.92)	0.556	47.00 (44.90–52.04)	49.00 (47.85–53.09)	0.116 ^M^
MDDB-EC right leg	51.00 (48.35–53.75)	51.00 (48.07–52.41)	0.992	53.00 (47.96–55.10)	51.00 (46.87–52.07)	0.551 ^M^

MAAP: mean deflection of the center of pressure of the feet from Point 0 in the direction of the *y*-axis in millimeters; EO: eyes opened; MAML: mean deflection of the center of pressure of the feet from Point 0 in the direction of the *x*-axis in millimeters; MDDB: median difference balance; EC: eyes closed. Significant differences between the results were obtained “before” and “after” MPA rehabilitation when *p* < 0.05. The letters L, M, and S indicate large, moderate, and small effect sizes, respectively.

**Table 4:** Correlations between results of stabilometric platform, MOCCA, and BDI test results among MS patients under the age of 65 with low and high fatigue.

Variable	ALL		LOW FATIGURE Group		HIGH FATIGUE Group	
	BDI Changes	MOCCA Changes	BDI Changes	MOCCA Changes	BDI Changes	MOCCA Changes
MAAP-EO changes	0.115	−0.06	0.104	−0.09	0.085	0.011
MAML-EO changes	0.273	0.065	0.288	0.006	−0.035	0.239
MDDB-EO left leg changes	0.093	−0.186	0.086	−0.25	−0.377	−0.047
MDDB-EO right leg changes	−0.093	0.186	−0.086	0.25	0.377	0.047
MAAP-EC changes	0.093	−0.161	0.093	−0.205	0.281	0.014
MAML-EC changes	0.233	−0.202	0.301	−0.246	0.267	0.331
MDDB-EC 1 left leg changes	−0.077	−0.149	−0.045	−0.123	0.088	0.136
MDDB-EC 2 right leg changes	0.073	0.14	0.045	0.123	0.373	−0.021

MAAP: mean deflection of the center of pressure of the feet from Point 0 in the direction of the *y*-axis in millimeters; EO: eyes opened; MAML: mean deflection of the center of pressure of the feet from Point 0 in the direction of the *x*-axis in millimeters; MDDB: median difference balance; EC: eyes closed.

**Table 5:** Correlations between results of stabilometric platform, GDS, and MMSE test results among MS patients over the age of 65 with low and high fatigue.

Variable	ALL		LOW FATIGURE Group		HIGH FATIGUE Group	
	GDS Changes	MMSE Changes	GDS Changes	MMSE Changes	GDS Changes	MMSE Changes
MAAP-EO changes	0.039	−0.048	0.041	−0.271	−0.371	0.372
MAML-EO changes	−0.071	0.056	−0.116	0.221	0.034	−0.034
MDDB-EO left leg changes	0.13	0.02	0.067	0.208	0.428	−0.429
MDDB-EO right leg changes	−0.13	−0.02	−0.067	−0.208	−0.429	0.429
MAAP-EC changes	0.115	−0.155	−0.107	0.066	0.676	−0.676
MAML-EC changes	0.277	−0.23	0.206	−0.251	0.446	−0.446
MDDB-EC 1 left leg changes	−0.035	0.134	−0.11	0.234	−0.135	0.135
MDDB-EC 2 right leg changes	0.186	−0.297	0.11	−0.234	0.541	−0.541

MAAP: mean deflection of the center of pressure of the feet from Point 0 in the direction of the *y*-axis in millimeters; EO: eyes opened; MAML: mean deflection of the center of pressure of the feet from Point 0 in the direction of the *x*-axis in millimeters; MDDB: median difference balance; EC: eyes closed.
